# Isolation and Identification of Lipid-Lowering Peptides from Sacha Inchi Meal

**DOI:** 10.3390/ijms24021529

**Published:** 2023-01-12

**Authors:** Kai Wang, Xiaofei Liu, Xuewu Zhang

**Affiliations:** College of Food Science and Engineering, South China University of Technology, Guangzhou 510641, China

**Keywords:** sacha inchi meal, pancreatic lipase, lipid-lowering peptides

## Abstract

Sacha inchi meal (SIM) is a by-product of sacha inchi (considered as a “super-food”) processing. In previous studies, we found that SIM protein hydrolysates exhibited pancreatic lipase inhibition activity. In this study, 10 bioactive peptides from those hydrolysates were identified. The top five peptides (NLYYKVV (NV-7), WWYVK (WK-5), WLLMWPYK (WK-8), EGLLMWPY (EY-8), and FPFFGYVWK (FK-9)) with strong pancreatic lipase inhibition activity had IC_50_ values of 34.01–246.50 µM, and displayed various inhibition types (mixed, non-competitive, and competitive type) by enzyme inhibition kinetics analysis. Fluorescence quenching analysis demonstrated that the interaction between the peptides and pancreatic lipase was mainly hydrogen bond and van der Waals force. The key residues involved in the peptide–enzyme interaction were determined by molecular docking. Moreover, the top two peptides were found to significantly inhibit fat accumulation and regulate lipid metabolism by alleviating the level of reactive oxygen species in HepG2 cells. Collectively, sacha inchi meal-derived peptides displayed potent lipid-lowering activity and could be used as materials of functional food.

## 1. Introduction

Obesity is essentially an excessive energy intake that is stored in the form of triacylglycerols in fatty tissue, which is a prevalent disease and has become a major problem in today’s society. Pancreatic lipase is a key lipase for lipid absorption throughout the body and is responsible for the hydrolysis of total dietary fats [1]. Its inhibition is efficient to prevent obesity, and is one of the most important methods for anti-obesity evaluation in vitro [2].

Protein hydrolysates are a complex mixture of peptides and amino acids. Food protein hydrolysates and peptides are considered as active ingredients in a functional food industry due to their numerous health benefits. In particular, many food-derived peptides exhibited anti-obesity activity. For example, Xiang et al. [3] revealed that six peptides (Glu-Glu-Ala-Ala-Ser-Leu-Arg, Phe-Arg, Arg-Asp-Arg, Ala-Pro-Tyr-Arg, Val-Arg, Asn-Leu-Leu-His-Arg) from sea buckthorn seed meal possessed anti-obesity effects. Esfandi et al. [4] found that the oat bran-derived peptide, SPFWNINAH, was the best lipase inhibitor (IC50 85.4 +/− 3 µM). Urbizo-Reyes et al. [5] showed that canary seed (*Phalaris canariensis* L.) peptides possessed in vitro antiobesity properties via the inhibition of pancreatic lipase. Wang et al. [6] screened five peptides, namely TF, EW, QWM, NIF, and AGY, from sesame proteins, which exhibited inhibitory activity on pancreatic lipase with IC50 values of 751 ± 75, 907 ± 91, 986 ± 170, 1044 ± 179, and 1183 ± 179 µM, respectively.

Usually, various methods are employed to obtain polypeptide components with better activity from protein hydrolysates. The widely used methods include the membrane separation techniques (ultrafiltration and nanofiltration), column chromatography, reversed-phase high-performance liquid chromatography, etc. [7]. Ultrafiltration affinity chromatography is used as a strategy to screen enzyme inhibitors from functional foods, its primary steps include: incubating the mixture with the target protein so as to bind the ligand in the mixture with the target protein, and then the protein–ligand complex is intercepted by the ultrafiltration membrane. Finally, the ligand is released from the denatured protein and analyzed by liquid chromatography, which avoids the complex steps of separation and purification. Using this method, 10 pancreatic lipase inhibitors and 14 α-glucosidase inhibitors in Moringa leaf were found [8,9]. In our research group, four α-glucosidase and two DPP-4 inhibitory peptides were identified from dark tea protein hydrolysates [10].

Sacha inchi (*Plukenetia volubilis* L.) is a local plant in the Amazon rainforest and is considered as a novel source of “super-food” [11]. Sacha inchi meal (SIM) is a by-product of sacha inchi oil production (annually over 150 tons in China), which contains a high protein content of 566–600 g/kg [12]. Now, these by-products are not effectively used in production systems, which cause environmental problems due to their easy decomposition [13]. To date, there are scarce reports about peptide generation from SIM. For example, the de-oiled and pressed cakes were hydrolyzed by crude papain and Calotropis proteases, and the antioxidant properties of the hydrolysates were evaluated [14]. The protein extracted from the pressed seed of sacha inchi was hydrolyzed by Alcalase (an endonuclease), Neutrase (an endonuclease), and Flavourzyme (belongs to exoenzyme and is mainly used to reduce or avoid the production of bitter peptides during hydrolysis) to obtain sacha inchi protein hydrolysates [15]. The study found that the hydrolysates obtained with Alcalase–Neutrase exhibited the lowest ACE (angiotensin-I converting enzyme) inhibition activity (IC50 value was 98 µg/mL) and a high ABTS antioxidant activity (1.19 µmol Trolox equivalent/mg). Similarly, pepsin, papain, and Flavourzyme were used to produce sacha inchi protein hydrolysates, and the pepsin-digested hydrolysate showed the highest antioxidant activities by DPPH (55.7%), FRAP (0.167 mmol Fe2+/g), and metal ion chelation activity (55.4%) [16]. The defatted sacha inchi seed was subjected to simulated gastrointestinal digestion, and the results indicated that the hydrolysate containing glutelin fraction showed the highest antioxidant activity, followed by albumin and globulin fractions [17]. However, the SIM-derived bioactive peptides were not identified.

In previous studies [18], we employed proteases to hydrolyze SIM protein, and found that the hydrolysates exhibited pancreatic lipase inhibition activity. The objective of this study was to identify the peptides in the hydrolysates responsible for the pancreatic lipase inhibition using ultrafiltration affinity chromatography in order to explore peptide–enzyme inhibition kinetics, and to investigate the lipid-lowering activity in HepG2 cells.

## 2. Results and Discussion

### 2.1. Peptides Identification and Pancreatic Lipase Inhibitory Activity

**Figure 1:**
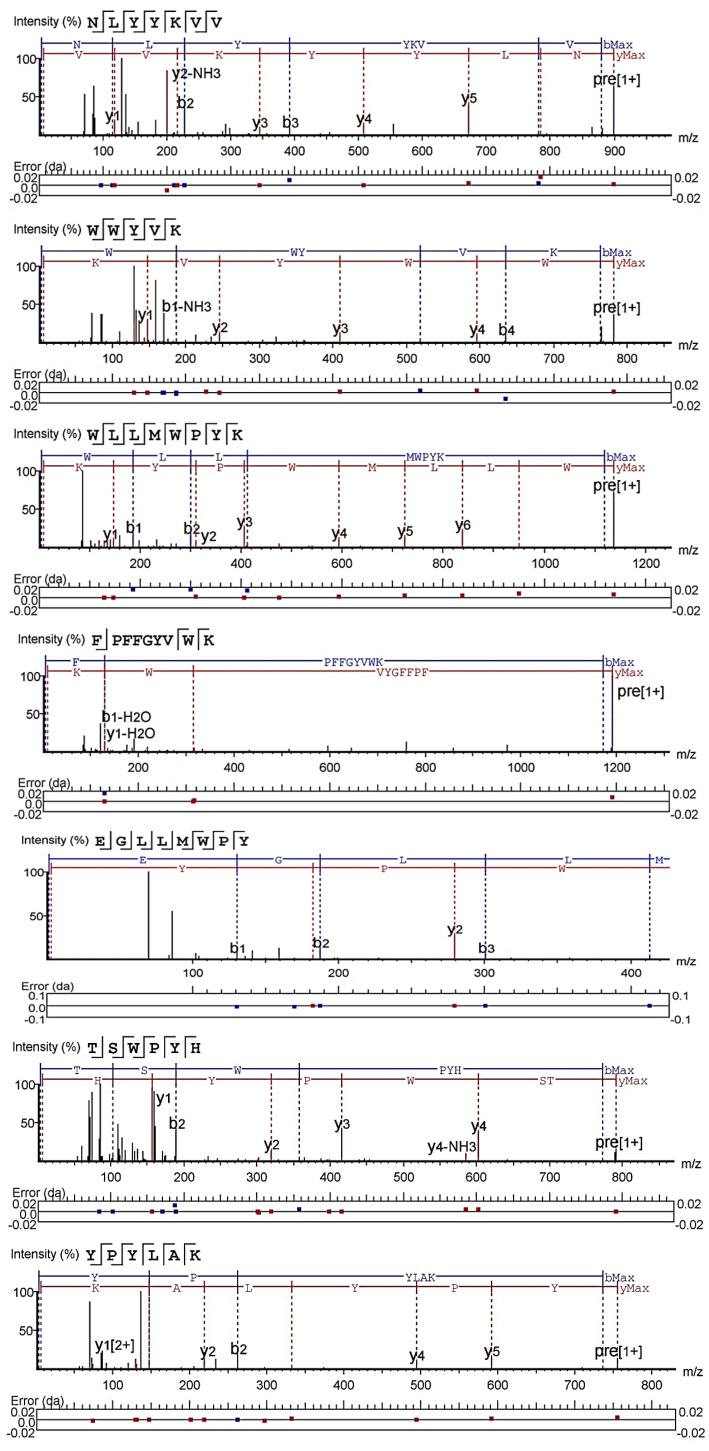

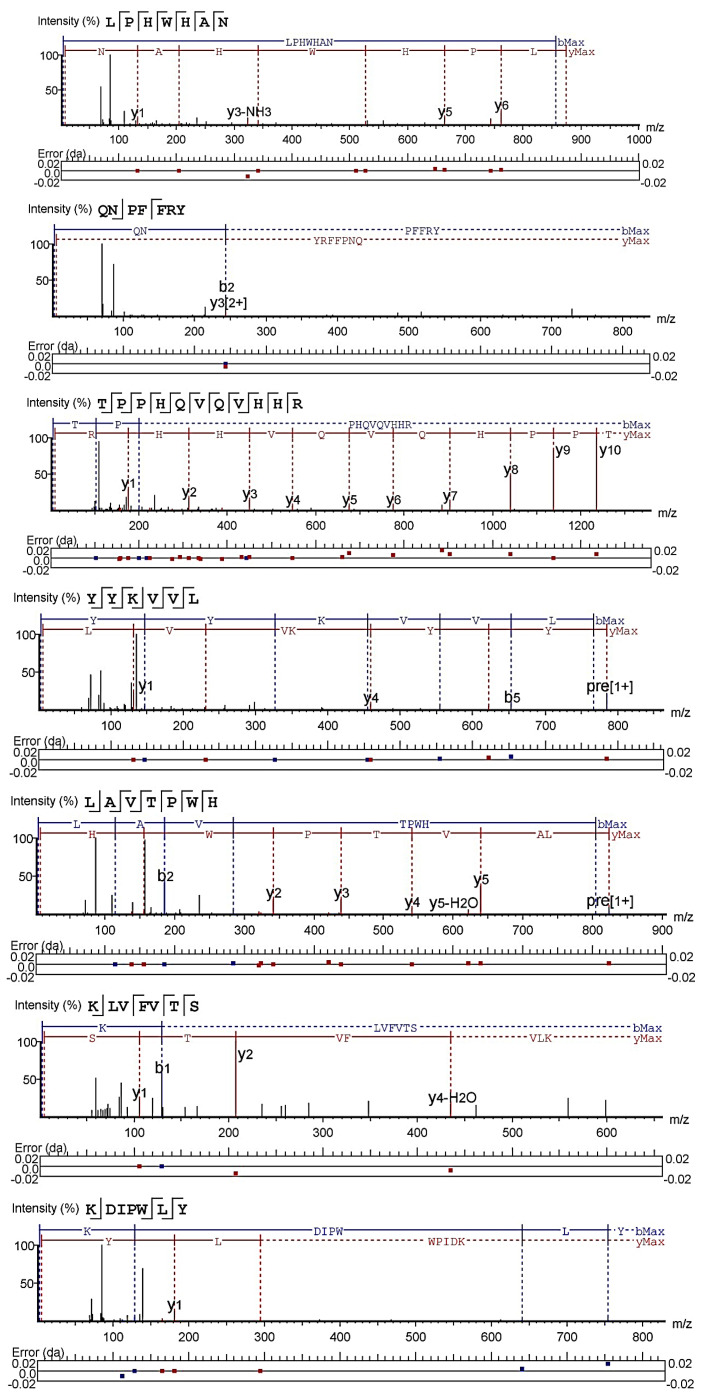
MS/MS diagram of pancreatic lipase inhibitory peptides identified from hydrolysates and the hydrolysates separated by ultrafiltration affinity chromatography.

The database search and de novo modules of Peaks Studio X software were used to analyze the mass spectrum data of four enzymatic hydrolysates, namely SPe, Spa, STr, and SBr. A total of 10 peptides were identified: NLYYKVV (NV-7), TSWPYH (TH-6), and YPYLAK (YK-6) from SPe; LPHWHAN (LN-7), WWYVK (WK-5), and QNPFFRY (QY-7) from Spa; WLLMWPYK (WK-8) and TPPHQVQVHHR (TR-11) from STr; and YYKVVL (YL-6) and LAVTPWH (LH-7) from SBr (Figure 1). Moreover, ultrafiltration affinity chromatography separation was performed on four enzymatic hydrolysates, namely SPe, Spa, STr, and SBr. The database search and de novo modules of Peaks Studio X software was also used to analyze their mass spectrum data, and four peptides were identified: FPFFGYVWK (FK-9) from SBr; EGLLMWPY (EY-8) from STr; KLVFVTS (KS-7) from SPe; and KDIPWLY (KY-7) from Spa (Figure 1).

**Figure 2:**
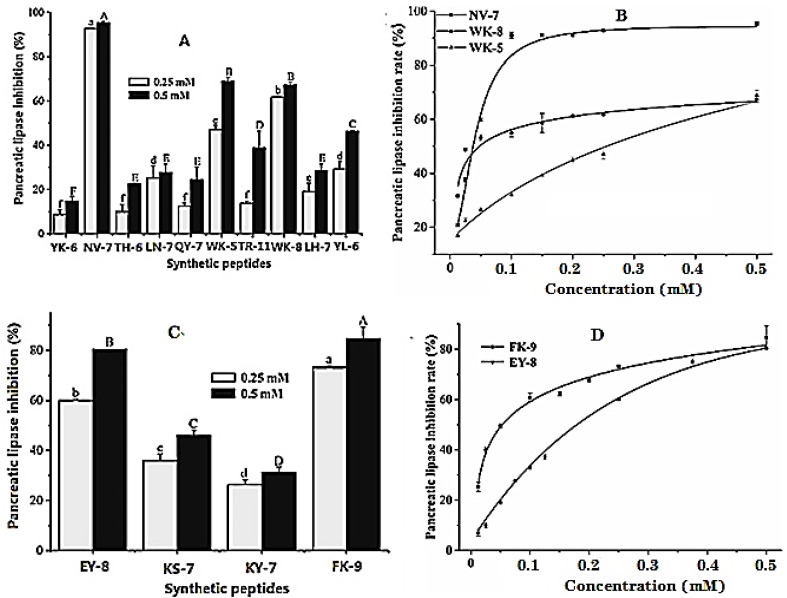
Inhibitory effects and IC50 calculation curves of synthetic peptides identified from hydrolysates (**A**,**B**) and the hydrolysates after ultrafiltration affinity chromatography (**C**,**D**) to pancreatic lipase. Different letters in the figure represent significant for different groups.

It can be seen from Figure 2A that the inhibitory activity of polypeptides on pancreatic lipase at the concentration of 0.25 and 0.5 mM gradually increased with the increase of concentration, and the order of inhibitory effect at a high concentration (0.5 mM) was: NV-7 > WK-5 > WK-8 > YL-6 > TR-11 > LH-7 > LN-7 > QY-7 > TH-6 > YK-6. The inhibitory percentages of the three polypeptides, namely NV-7, WK-5, and WK-8 with the best activity on pancreatic lipase were 95.42 ± 0.60%, 68.93 ± 1.80%, and 67.33 ± 1.10%, respectively, at the concentration of 0.5 mM. Figure 2C describes the inhibitory effect of synthetic peptides screened by ultrafiltration affinity chromatography on pancreatic lipase. With the increase of concentration, the inhibitory activity of the four synthetic peptides on pancreatic lipase gradually increased, and the order of inhibitory effect was: FK-9 > EY-8 > KS-7 > KY-7. When the concentrations of FK-9 and EY-8 were 0.5 mM, the inhibitory rates were 84.63 ± 4.67% and 80.47 ± 0.11%, respectively. The activities were significantly higher than those of the active peptides directly screened from enzymatic hydrolysates (Figure 2A), indicating that ultrafiltration affinity chromatography was reliable and had a high hit rate; it can be used for rapid screening of pancreatic lipase inhibitory peptides.

The dose–activity relationship (Figure 2B,D) showed that the IC50 values of five peptides with good inhibitory activity against pancreatic lipase ranged from 34.01 to 246.50 μM. The synthetic peptide NV-7 with the best activity was selected from the hydrolysates, and the IC50 value was 34.01 μM. The synthetic peptide AQ-7 with the best activity screened by ultrafiltration affinity chromatography had an IC50 value of 55.13 μM.

### 2.2. Inhibition Kinetics of Pancreatic Lipase

The inhibition kinetics of pancreatic lipase for the three synthetic peptides with the best activity was analyzed by a double reciprocal Lineweaver–Burk diagram to evaluate the type of pancreatic lipase inhibition. As shown in Figure S1A, under the action of NV-7 with different concentrations, all fitting curves of the Lineweaver–Burk diagram intersected in the third quadrant, and the increase of slope gradually decreased with the increase of NV-7 concentration, indicating that the inhibition of NV-7 on pancreatic lipase was a mixed mode. The Michaelis constant Km is 1.67 mM ± 0.07 mM, and the equilibrium constants Ki and Ki’ were 77.33 ± 7.65 μM/(ms^2^ FLU^2^) and 7.8 ± 0.77 μM/(ms^2^ FLU^2^), respectively (Table S1). When Ki > Ki′, the inhibition type was a mixed inhibition dominated by competition. NV-7 formed an enzyme inhibitor complex with the active site of the enzyme, which was more stable.

It can be seen from Figure S1B that under different WK-5 concentrations, Lineweaver–Burk double reciprocal curves of pancreatic lipase substrate concentration and reaction rate intersected in the second quadrant, and the inhibition type was mixed-type inhibition. The Michaelis constant Km was 0.67 ± 0.01 mM, and the equilibrium constants Ki and Ki′ were 577.60 ± 25.69 μM/(ms^2^ FLU^2^) and 1225.28 ± 55.20 μM/(ms^2^ FLU^2^), respectively (Table S1). When Ki < Ki′, the mixed inhibition type was mainly non-competitive, and the enzyme substrate inhibitor complex formed by WK-5 and the non-active site of the enzyme was more stable. It can be seen from Figure S1C that under the inhibition of WK-8, the fitting curve drawn by the reciprocal of substrate concentration and the reciprocal of reaction rate intersected at the negative half axis of the *X* axis. WK-8 was a typical non-competitive inhibitor of pancreatic lipase. WK-8 could form a stable complex with the enzyme substrate to inhibit the enzyme activity. It can be calculated that Km is 0.46 ± 0.02 mM, and the equilibrium constant Ki was 191.91 ± 25.33 μM/(ms^2^ FLU^2^). In Figure S1D, under the action of the concentration gradient of EY-5, the substrate concentration of pancreatic lipase and the Lineweaver–Burk double reciprocal curve of reaction speed intersected at the third quadrant, and the inhibition type was mixed inhibition; Km was 0.15 ± 2.71 × 10^−3^ mM and the equilibrium constants Ki and Ki’ were 578.30 ± 58.12 μM/(ms^2^ FLU^2^) and 161.47 ± 16.22 μM/(ms^2^ FLU^2^), respectively (Table S1). When Ki > Ki, the inhibition type was a mixed inhibition dominated by competition, and WK-8 formed an enzyme inhibitor complex with the active site of the enzyme, which is more stable. Similarly, under the inhibition of FK-9, the double reciprocal Lineweaver–Burk fitting curve intersected at the positive half axis of the Y axis, indicating that FK-9 was a competitive inhibitor of pancreatic lipase, which could form a stable complex with the enzyme and prevent the substrate from entering the enzyme active site. It can be calculated that under the action of FK-9, the Km of pancreatic lipase was 0.22 ± 0.01 mM, and the equilibrium constant Ki was 80.94 ± 2.85 μM/(ms^2^ FLU^2^).

### 2.3. UV Spectroscopic Analysis of Synthetic Peptide and Pancreatic Lipase

Figure S2 showed that under the action of different concentrations of NV-7 and FK-9, the maximum absorption peak wavelength of pancreatic lipase UV spectrum had a slight red shift, moving from 202.5 nm to 204 nm and 203.5 nm, respectively, indicating that NV-7 and FK-9 formed hydrogen bonds or conjugation with pancreatic lipase, making the maximum absorption wavelength move to the long wave direction. In a word, NV-7 and FK-9 had an obvious interaction with pancreatic lipase.

### 2.4. Fluorescence Quenching Analysis of Pancreatic Lipase

Fluorescence quenching spectrum is an important tool to study the interaction between small molecules and proteins. The aromatic amino acid residues (tyrosine, phenylalanine, and tryptophan) contained in proteins have intrinsic fluorescence. When the protein conformation changes under the action of inhibitors, fluorescence quenching will occur. The fluorescence quenching spectrum of pancreatic lipase by NV-7 and FK-9 was shown in Figure S3. The fluorescence intensity of pancreatic lipase showed dose-dependent quenching with the increase of the concentration of NV-7 and FK-9, indicating that NV-7 and FK-9 interacted with pancreatic lipase, resulting in changes in the conformation of the enzyme. According to Figure S3A, the relationship between F0/F and [NV-7] showed that the linear concentration of fluorescence quenching of pancreatic lipase was 2–30 mM. In Table S2, as the temperature increases from 298 K to 310 K, the quenching constant Ksv of pancreatic lipase by NV-7 decreased from 4.62 × 103 L/mol to 3.27 × 10^3^ L/mol, Kq decreased from 4.62 × 10^11^ mol/(L·s) to 3.27 × 10^11^ mol/(L·s), both greater than the maximum fluorescence dynamic quenching rate constant 2 × 10^10^ mol/(L·s), and the quenching constant Ksv gradually decreased with the increase of temperature, indicating that the increase of temperature was not helpful to the combination of NV-7 and pancreatic lipase. Thus, the fluorescence quenching of pancreatic lipase by NV-7 was a static quenching process [19], and the results are shown in Table S2. With the increase of temperature, the number of binding sites between NV-7 and pancreatic lipase gradually decreased from 2 to 1, and Ka gradually decreased from 10.66 × 10^3^ L/mol to 4.92 × 10^3^ L/mol, indicating that there were two binding sites between NV-7 and pancreatic lipase, and the binding stability of one site to NV-7 was negatively correlated with temperature, which was converted from static quenching to dynamic quenching. The results were consistent with the increase of the slope of the F0/F and [NV-7] curve at a high concentration. With the increase of temperature, ΔG*○* increased from −23.20 kJ/mol to −22.15 kJ/mol, which was also verified by the gradual increase of the free energy of spontaneous reaction; ΔH*○* < 0 and ΔS*○* < 0 indicated that the spontaneous reaction was driven by enthalpy change, and the interaction between NV-7 and pancreatic lipase was mainly hydrogen bond and van der Waals force [20]. According to Figure S3B, the fluorescence quenching F0/F of pancreatic lipase had a linear relationship with [FK-9]. With the increase of temperature, Ksv increased from 6.95 × 10^3^ L/mol to 11.09 × 10^3^ L/mol, Kq increased from 6.95 × 10^11^ mol/(L·s) to 11.09 × 10^11^ mol/(L·s), both greater than the maximum fluorescence dynamic quenching rate constant 2 × 10^10^ mol/(L·s), so fluorescence quenching was mainly caused by the FK-9-pancreatic lipase complex formed by static quenching, and the gradual reduction of Ksv was the characteristic of intermolecular hydrophobic interaction under static quenching [21]. The binding site of FK-9-and pancreatic lipase was about 1. With the increase of temperature, Ka gradually changed from 3.56 × 10^3^ L/mol to 4.89 × 10^3^ L/mol, FK-9-pancreatic lipase complex was more and more stable, and the results were consistent with thermodynamic parameters; ΔG*○* decreased from −20.21 kJ/mol to −21.84 kJ/mol, and the spontaneous reaction was more consistent with the increase of temperature. $\Delta H^{○}$ > 0 and $\Delta S^{○}$ > 0 showed that hydrophobic and electrostatic interactions were the main driving forces for FK-9 to bind to pancreatic lipase [12].

### 2.5. Molecular Docking

**Figure 3:**
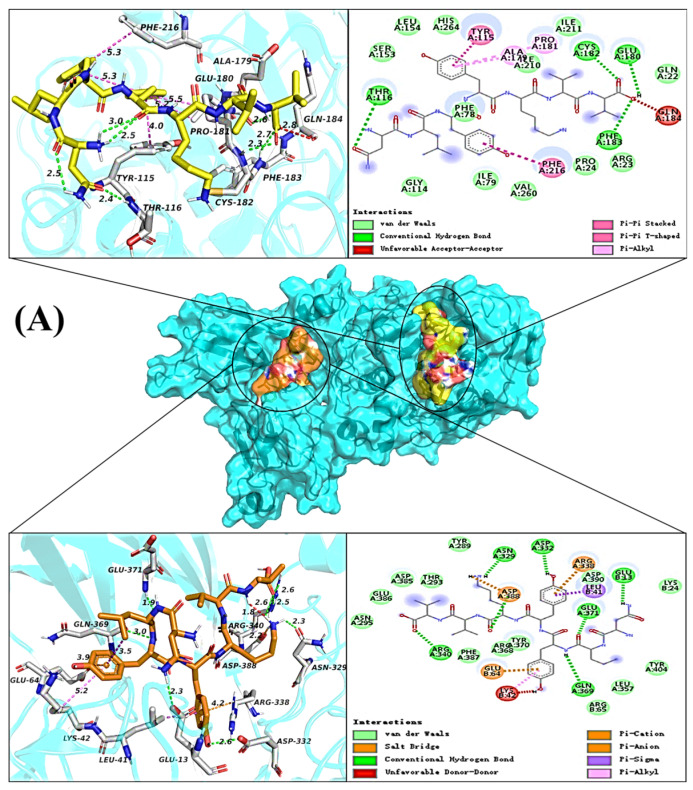

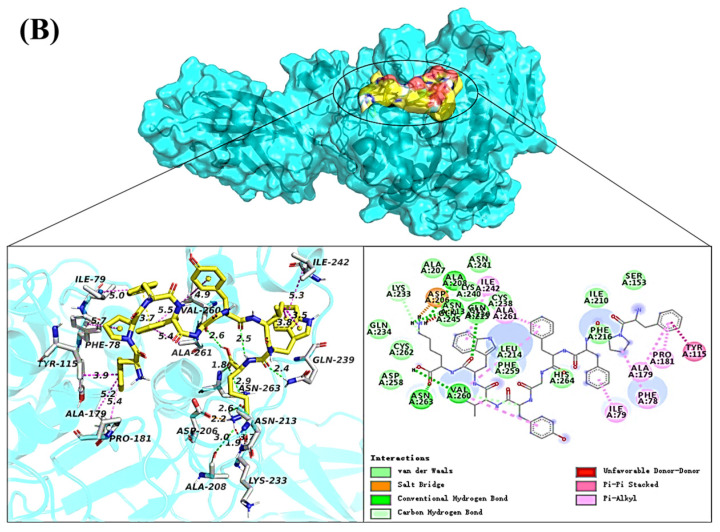
Molecular docking analysis of pancreatic lipase with peptides (**A**) NV-7; (**B**) FK-9.

According to the inhibition kinetics analysis, the inhibition type of NV-7 on pancreatic lipase was a mixed inhibition dominated by competition, and it had two binding sites with pancreatic lipase. The interaction between NV-7 and pancreatic lipase at two sites is shown in Figure 3A. The molecular docking energies of NV-7 with pancreatic lipase active pocket (right pocket) and non-active pocket were −7.0 kcal/mol and −7.6 kcal/mol, respectively. At the active pocket, NV-7 can form four hydrogen bonds with amino acid residues Thr116, Cys182, Glu180, and Phe183, and there were hydrophobic interactions with Tyr115, Pro181, Ala179, Gln184, and Phe216. Van der Waals force was the main force between NV-7 and pancreatic lipase catalytic sites Ser153, Asp177, and His264, indicating that hydrogen bonds and hydrophobic interactions enhanced the stability of NV-7 and pancreatic lipase complex at this binding site; the van der Waals force between NV-7 and the catalytic site hindered the formation of intermediates between the substrate and the enzyme. At the non-active pocket, NV-7 can form seven hydrogen bonds with the pancreatic lipase A chain: Asn329, Asp322, Gln369, Arg340, Asp388, Glu371 and B chain: Glu13, and there were hydrophobic interactions with the A chain Asp388, Arg338 and B chain: Leu41, Glu64, Lys42. The fluorescence quenching spectrum analysis of pancreatic lipase showed that NV-7 bound to pancreatic lipase ΔH*○* < 0, ΔS*○* < 0∆, the interaction between molecules was mainly van der Waals force and hydrogen bond, and the two results were consistent.

The binding of FK-9 to pancreatic lipase at the catalytic site was shown in Figure 3B. FK-9 had only one binding site with pancreatic lipase, and the molecular docking energy was −8.4 kcal/mol. At the active pocket, FK-9 can form six hydrogen bonds with amino acid residues Ala208, Asn213, Gln245, Gly215, Asn263, and Val260, and had hydrophobic interaction with Ile242, Ala261, Asn213, Val260, Ile79, Phe78, Ala179, Pro181, and Tyr115, electrostatic interaction with Asp206, and were consistent with fluorescence quenching $\Delta H^{○}$ > 0, $\Delta S^{○}$ > 0. Hydrophobic and electrostatic interactions were the main driving forces of FK-9 binding to pancreatic lipase.

### 2.6. Effect of Synthetic Peptides on HepG2 Viability

**Figure 4:**
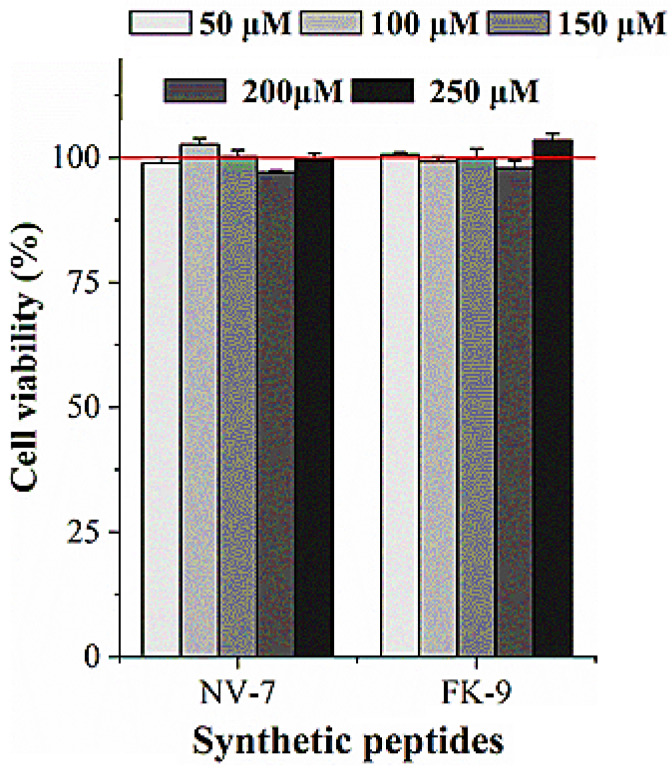
Effect of synthetic peptides on HepG2 viability.

The effect of synthetic peptides on the proliferation of HepG2 cells was detected by the MTT method. It can be seen from Figure 4 that at the concentration of the synthetic peptides 50 μM–250 μM, the activity of HepG2 cells was not significantly reduced, indicating that the peptides were basically non-toxic to HepG2 cells.

### 2.7. Effect of Synthetic Peptides on Fat Accumulation in HepG2 Cells

**Figure 5:**
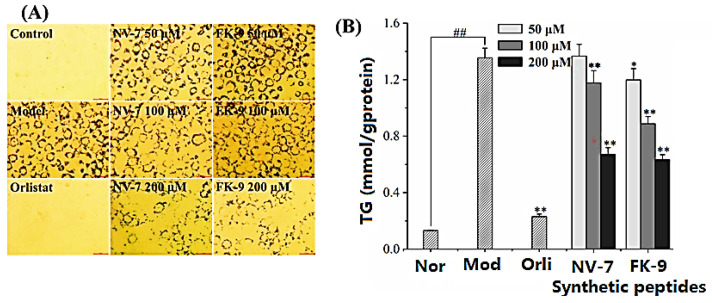
Effect of synthetic peptides on intracellular oil accumulation and triglyceride content of HepG2 (**A**) oil red O staining; (**B**) triglyceride content.

The effects of pancreatic lipase inhibitory peptides NV-7 and FK-9 on fat and triglyceride accumulation in HepG2 are shown in Figure 5. The oil red O staining of HepG2 cells after induction in Figure 5A showed that compared with normal cells, the model group showed that there was a large amount of oil accumulation in HepG2 cells induced by oleic acid, and the positive control orlistat can significantly reduce the oil accumulation in cells. Under the effect of different concentrations of synthetic peptides, the number of lipid droplets decreased to varying degrees, and with the increase of peptide concentration, the cell coloring became lighter, indicating that the inhibition of HepG2 cell fat accumulation by peptides was positively correlated with the concentration. Under the action of polypeptides, the TG content in HepG2 cells is shown in Figure 5B. The four polypeptides have similar effects on the TG content in HepG2 cells, which was consistent with the result of oil red O staining. Compared with the model group (1.36 ± 0.07 mmol/g protein), at 100 μM and 200 μM, synthetic peptides can significantly reduce intracellular TG levels. Under the action of 200 μM of synthetic peptide, the content of TG was 0.67 ± 0.05 mmol/g protein (NV-7) and 0.65 ± 0.01 mmol/g protein (FK-9), respectively. In conclusion, pancreatic lipase inhibitory peptides NV-7 and FK-9 can significantly inhibit fat accumulation in HepG2 cells and regulate lipid metabolism in HepG2 cells.

### 2.8. Effect of Synthetic Peptides on Reactive Oxygen Species in Oleic Acid-Induced HepG2 Cells

**Figure 6:**
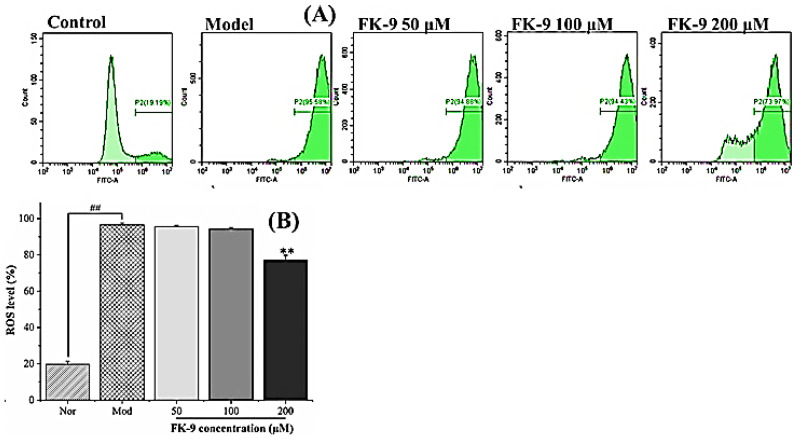
Effect of synthetic peptides on intracellular ROS content of HepG2. ## very significant (*p* < 0.01) comparison between normal group and model group, ** very significant (*p* < 0.01) comparison between peptide group and model group. (**A**) Flow cytometry (FITC-A) analysis of intracellular ROS, (**B**) Intracellular ROS content calculated by flurescent intensity in (**A**).

The effect of pancreatic lipase inhibitory peptide FK-9 on the level of HepG2 reactive oxygen species induced by oleic acid is shown in Figure 6. The level of reactive oxygen species in HepG2 induced by oleic acid increased significantly. The level of intracellular reactive oxygen species in HepG2 in the normal group was only 19.67 ± 1.71%, while that in the model group was as high as 96.52 ± 1.03%. Polypeptide FK-9 at a low concentration (50–100 μM) had no significant effect on the level of reactive oxygen species. When the concentration was 200 μM, the production of reactive oxygen species was significantly inhibited (*p* < 0.01), and the level of reactive oxygen species decreased to 77.03 ± 2.77%. Studies have shown that the fat accumulation of HepG2 cells induced by oleic acid was significantly related to intracellular oxidative stress [22]. Hence, FK-9 can alleviate oxidative stress by reducing the level of HepG2 reactive oxygen species induced by oleic acid, thereby reducing HepG2 fat accumulation.

Pancreatic lipase is a key lipase for lipid absorption throughout the body and is responsible for the hydrolysis of total dietary fats, and its inhibition is efficient to prevent obesity. In previous studies, many peptides were identified from plant-derived proteins. From acidic protease hydrolysate of globin protein, Kagawa et al. [23] identified a peptide VVYP with an inhibitory effect on hypertriglyceridemia. Fan et al. [24] identified four lipid-lowering peptides, namely NALKCCHSCPA, LNNPSVCDCDCMMKAAR, NPVWKRK, and CANPHELPNK, from Spirulina platensis protein, which exhibited inhibitory effects on 3T3-L1 preadipocytes proliferation (32.29–60.08%). In particular, NPVWKRK and CANPHELPNK also significantly decreased the accumulation of triglyceride at 600 μg/mL (*p* < 0.05), with 23.7% and 19.5% of inhibition on triglyceride production, respectively, compared with the control. Zhang et al. [25] identified a novel lipid inhibition peptide Leu-Leu-Val-Val-Try-Pro-Trp-Thr-Gln-Arg (PP1) (MW 1274.53 Da) from Chlorella pyenoidose, which exhibited a good inhibitory effect against porcine pancreatic lipase (47.95%) at 200 µg/mL, and decreased the accumulation of intracellular triacylglycerol (27.9%, 600 µg/mL) in 3T3-L1 cells. From oat protein hydrolysates, Esfandi et al. [4] identified SPFWNINAH as the lipase inhibitor (IC50 85.4 +/− 3 µM), Using the CABS-dock computational model, they predicted that interactions between the peptide and pancreatic lipase residues of Ser (153), His (264), and Asp (177) were important for the inhibition. Wang et al. [6] discovered five pancreatic lipase inhibitory peptides, namely TF, EW, QWM, NIF, and AGY from sesame proteins with IC50 values of 751–1183 µM. In this study, five peptides (NV-7, WK-5, WK-8, EY-8, FK-9) with strong pancreatic lipase inhibition activity had IC50 values of 34.01, 246.50, 52.79, 171.00, and 55.13 µM, respectively. Compared with the results in the literature, the peptides identified in the present study displayed strong activity; however, further in vivo study and acting mechanisms are highly required in the future.

## 3. Materials and Methods

### 3.1. Enzymatic Hydrolysis

Ultrasonic alkali-soluble acid precipitation was employed to extract the protein of sacha inchi meal protein. Specifically, sacha inchi seed meal was crushed and passed through a 100-mesh sieve. Then, the powder was mixed with petroleum ether at a ratio of 1:5 (g/mL), stirred at 50 °C for 6 h, centrifuged at 1253 g for 30 min, and repeated three times. The precipitate was collected, dried at 60 °C, and stored at −20 °C. The defatted powder of SIM was dispersed in ultrapure water at a ratio of 1:10 (g/mL), the pH was adjusted to 10.0 by dropping 1 M NaOH, and after ultrasonic treatment (Scientz-IID, Ningbo, China) at 400 W for 30 min at 50 °C, the mixture was stirred in a water bath for 2 h, and centrifuged (H2050R, Xiangyi, China) at 1253 g for 30 min. Then, the supernatant was taken, and the residue was treated twice according to the above process. Combining the supernatant, and adding 1 M HCl to adjust the pH to 4.5, the protein was precipitated at 4 °C overnight for 15 min, and centrifuged at 1253 g. Then, the precipitated protein was washed with 5 times ultrapure water 3 times, adjusting the pH to 7.0. The protein was redissolved, subjected to a 5000 Da dialysis bag for 48 h to remove salt, freeze-dried (MODULYOD type, Thermo Scientific, Guangzhou, China), and stored at −20 °C.

The extracted protein (10 g/mL) was subjected to enzymatic hydrolysis under the optimal conditions of four enzymes (enzyme to substrate ratio (the mass of enzyme related to the mass of SIM), temperature, pH, time) according to the literature: for pepsin (5%, 37 °C, pH = 2, 4 h), the hydrolysate was SPe; for papain (5%, 55 °C, pH = 7, 4 h), the hydrolysate was SPa; for trypsin (5%, 37 °C, pH = 8, 4 h), the hydrolysate was STr; for bromelain (5%, 55 °C, pH = 7, 4 h), the hydrolysate was SBr. The hydrolysis details referred to [18]. After enzymolysis, adjusting the pH = 7, being boiled in boiling water for 10 min to inactivate the enzyme, centrifuged at 5013 g at 4 °C for 10 min, the supernatant was collected, freeze-dried, and stored at −20 °C. The BCA method was used to determine the content of polypeptide in hydrolysates of SIM.

### 3.2. Separation of Ultrafiltration Affinity Chromatography

The ultrafiltration affinity chromatography was used based on Fang et al. [9]. Specifically, pancreatic lipase was prepared into 2 mg/mL solution with 0.0013 mol/L (pH = 7.9) of Tris HCl buffer solution (containing 0.15 mol/L NaCl and 0.0013 mol/L CaCl_2_), centrifuged at 1253 g for 5 min, and then the supernatant was taken for standby. Four hydrolysates, namely SPe, SPa, STr, and SBr, were prepared into 5 mg/mL solutions. Taking 100 μL of the sample solution and 100 μL of the enzyme solution, we put them into a 0.4 mL of 30 kD molecular weight ultrafiltration tube, incubated them at 37 °C for 30 min, centrifuged them at 7833 g for 10 min, after which the filtrate A1 was collected, which was a component not binding to pancreatic lipase. After cleaning with a 200 μL buffer solution, being centrifuged at 7833 g for 10 min, repeating this 3 times, and combining the filtrate, then, 200 μL of acetonitrile solution was added to the ultrafiltration tube to elute the components binding with pancreatic lipase. These were then mixed evenly, stood still for 10 min, centrifuged at 7833 g for 10 min, and the filtrate B1 was collected. This was repeated 3 times, before we combined the filtrate. We mixed the pancreatic lipase solution, boiled, and inactivated at the same concentration with the sample solution. Then, we repeated the above steps, combined the filtrates, and A2 and B2 were obtained.

### 3.3. Mass Spectrometry Identification

UPLC (reversed phase ultra-performance liquid chromatography)-ESI-MS/MS was performed with the filtrates A1, B1, A2, and B2. Specifically, a Thermo Fisher (Guangzhou, China) ScientificTMQExactive combined four-stage Orbitrap mass spectrometer was used. The chromatographic column was BEH C18 (1.7 μm, 2.1 × 100 mm); the detector was an ultraviolet detector. The chromatographic conditions were as below: injection volume: 20 μL, mobile phase A: water containing 0.1% formic acid; mobile phase B: acetonitrile containing 0.1% formic acid, detection wavelength: 220 nm. The elution conditions were as follows: flow rate: 0.3 mL/min, mobile phase B elution from 0–40% gradient in 0–30 min, and mobile phase B elution at 5% isocratic in 30–35 min. The mass spectrometry conditions include: positive ion mode, Sheath gas flow rate: 45 L/min, Aux gas flow rate: 15 L/min, Sweep gas flow rate: 2 L/min, atomization voltage: 3.50 kV, capillary temperature: 320 °C, S-lens RF level: 55.0, Aux gas heater temp: 350 °C, and the scanning range was 200–3000 *m*/*z*. Then, a database search and de novo modules of Peaks Studio X software were employed to analyze the mass spectrum data. The protein database was the UniProt protein database (https://www.uniprot.org/) (accessed on 20 May 2022) for Plukenetia Volubilis species. The search parsing parameters were parent mass error tolerance 20.0 ppm, fragment mass error tolerance 0.1 Da, and Max missed cleavages 100. The de novo resolution parameters were parent mass error tolerance 20.0 ppm and fragment mass error tolerance was 0.1 Da. Based on −10lgp > 15 and de novo > 50, the peptide sequences were identified.

### 3.4. Computer Screening

Using PyMOL software to dewater the protein and delete the original ligand, the identified peptides were docked with pancreatic lipase (PDB ID: 1ETH) by HPEPDOCK (http://huanglab.phys.hust.edu.cn/hpepdock/) (accessed on 20 May 2022), analyzing docking energy and selecting peptides for further solid-phase synthesis.

### 3.5. Solid-Phase Synthesis of Peptides

The selected peptides were synthesized by the standard solid-phase synthesis method (Top-Peptide Co., Ltd., Shanghai, China). HPLC-ESI-MS was used to identify the target peptide and determine the content of synthetic peptide (purity > 95%). Chromatographic conditions: chromatographic column was C18 (4.6 × 250 mm, 5 μM), mobile phases A and B were water and acetonitrile containing 0.1% trifluoroacetic acid, respectively, with an injection volume of 20 μL, flow rate of 1.0 mL/min, and detection wavelength of 214 nm. Mass spectrometry conditions: positive ion mode, nitrogen flow rate: 1.5 L/min, CDL: −20.0 V, CDL temperature: 250 °C, Block temperature: 400 °C.

### 3.6. Pancreatic Lipase Inhibitory Activity

Trypsin (10 mg) was dissolved in 1 mL of Tris HCl buffer solution (pH = 7.9, 0.0013 mol/L, containing 0.15 mol/L NaCl and 0.0013 mol/L CaCl_2_), centrifuged at 4 °C, 1253 g for 5 min. The sample was diluted to an appropriate concentration with Tris HCl buffer. The sample solution (25 μL), pancreatic lipase solution (25 μL) and 4-MUO (50 μL, 20 μM) were added to a 96-well plate, after 20 min incubation at 25 °C, 100 μL of sodium citrate solution (0.1 M, pH = 4.2) was added to terminate the reaction. The fluorescence intensity was measured at excitation wavelength λex = 320 nm and emission wavelength λem = 450 nm. A buffer (25 μL) and enzyme solution (25 μL) were added for the control group. A sample solution (25 μL) and buffer (25 μL) were added for background group. Each group of experiments was repeated 3 times. The calculation formula of pancreatic lipase inhibition rate was as follows:

(1) $$\mathrm{Lipase}\;\mathrm{inhibition}\;\mathrm{rate}\;\left(\%\right)=\left(1-\frac{{\mathrm{FLU}}_{\mathrm{sample}}-{\mathrm{FLU}}_{\mathrm{background}}}{{\mathrm{FLU}}_{\mathrm{negative}\;\mathrm{control}}-{\mathrm{FLU}}_{\mathrm{negative}\;\mathrm{background}}}\right)\times 100$$

Among them, FLU_sample_ was the light absorption value of the experimental group; FLU_background_ was the light absorption value of the sample background group; FL_Unegative_ control was the absorbance value of the control group without samples; and FLU_negative_ background was the absorbance value of the background of the control group.

### 3.7. Inhibition Kinetics of Enzyme

Synthetic peptides were diluted with Tris HCl buffer to the appropriate concentration, and pancreatic lipase was prepared with a concentration of 40 μg/mL reaction solution, and the substrate 4-MUO was diluted to the concentrations of 50, 100, 150, 200, and 250 μM solution. In total, a 25 μL sample solution of different concentrations, a 25 μL enzyme solution, and 50 μL substrates with different concentrations were added to the 96-well plate. The fluorescence value FLU was immediately measured at the excitation wavelength λex = 320 nm and emission wavelength λem = 50 nm, every 1 min within 20 min, and each group of experiments was repeated 3 times. The slope of the “fluorescence value-time” linear fitting curve represented the reaction rate of the enzyme in the presence of the sample. The Lineweaver–Burk double reciprocal diagram was drawn to analyze the inhibition type of synthetic peptides on enzymes.

### 3.8. Ultraviolet Spectrum Scanning Determination

Pancreatic lipase was diluted to 2.0 × 10^−6^ mol/L with tris HCl buffer solution. An enzyme solution (1 mL) was added to the quartz cuvette, and then the peptide solutions (10 μL) of different concentrations were added to the cuvette step-by-step. After 5 min of equilibrium, the absorbance value of the enzyme and the solution were measured at a 190–400 nm wavelength after each sample addition was recorded at 298 K (UV2300II, Tianmei, China).

### 3.9. Fluorescence Quenching Assay of Pancreatic Lipase

An enzyme (150 μL) was added to a black 96-well plate, and the final concentration was 2.0 × 10^−6^ mol/L. Then, 50 μL polypeptide solutions of different concentrations (final concentration: 0, 2, 5, 10, 20, 30, 40, 50 μM) were added to the perforated plate, and the fluorescence spectra (Neo2 Hybrid, BioTek, Guangzhou, China) of the mixture was measured at 298, 304, and 310 K (25, 31, 37 °C), respectively. The excitation wavelength was λex = 285 nm, the scanning range of emission wavelength λem was 285–500 nm, and the step length was 2 nm. In order to eliminate the influence of the internal filtering effect, all fluorescence values were adjusted according to the following formula:

(2) Fc=Fme(A1+A2)2

where *F_c_* and *F_m_* represent the updated and detected fluorescence intensity, and *A*_1_ and *A*_2_ represent the absorbance values of the polypeptide at the excitation wavelength (280 nm) and emission wavelength, respectively. All solution concentrations correspond to fluorescence measurements.

The quenching type of synthetic peptide and pancreatic lipase can be judged by the Stern–Volmer equation:

(3) $$\frac{F_{0}}{F}=K_{SV}\left[Q\right]+1=K_{q}\tau_{0}\left[Q\right]+1$$

where *F*_0_ and *F* are the fluorescence intensity of the system in the absence and presence of quenchers, respectively; *K_sv_* is the Stern–Volmer quenching constant; [*Q*] is the concentration of quencher; *K_q_* is the rate constant of bimolecular fluorescence quenching; τ_0_ is the average fluorescence lifetime of the substance in the absence of quencher, and the average fluorescence lifetime of general biological macromolecules is 1 × 10^−8^ s.

When the phosphor is a biological macromolecule, the maximum fluorescence dynamic quenching rate constant is 2 × 1010 mol/L s, If *K_q_* > 2 × 1010 mol/L s, and it belongs to static quenching. If *K_q_* < 2 × 1010 mol/L s, it belongs to dynamic quenching. When *K_q_* cannot be calculated, the quenching type can be judged according to the law of *K_sv_* changing with temperature: dynamic quenching mainly depends on diffusion, and the diffusion coefficient increases with the increase of temperature, and *K_sv_* increases. In static quenching, the stability of the complex decreases, the degree of static quenching decreases, and *K_sv_* decreases with the increase of temperature. Therefore, when the *K_sv_* values at different temperatures in the same system become smaller with the increase of temperature, it belongs to static quenching. When *K_sv_* increases with the increase of temperature, it belongs to dynamic quenching.

When the quenching type is static quenching, the binding constant *K_a_* and binding site n of the quenching agent and the phosphor can be calculated by the following formula:

(4) $$\mathrm{lg}\frac{\left(F_{0}-F\right)}{F}=lgK_{a}+nlg\frac{1}{\left[Q_{0}\right]-\left[P_{0}\right]\frac{\left(F_{0}-F\right)}{F_{0}}}$$

[*Q*_0_] and [*P*_0_] are the total concentrations of quenching agent and fluorescent agent, respectively; *K_a_* is the binding constant; and n is the number of intermolecular binding sites between fluorescent agent and quencher.

By linear regression $\mathrm{lg}\frac{\left(F_{0}-F\right)}{F}$ and $lg\frac{1}{\left[Q_{0}\right]-\left[P_{0}\right]\frac{\left(F_{0}-F\right)}{F_{0}}}$, the slope and intercept are the logarithm of the number of binding sites n and the binding constant Ka between the two molecules, respectively.

The thermodynamic parameters of the binding reaction between quencher and phosphor were used to analyze the type of molecular interaction between them. Thermodynamic parameters can be calculated by Van ’t Hoff equation:

(5) $$\Delta G^{○}=RlnK_{a}=\Delta H^{○}-T\Delta S^{○},\;lgK_{a}=-\frac{\Delta H^{○}}{2.303RT}+\frac{\Delta S^{○}}{2.303R}$$

*K_a_* is the binding constant of quenching agent and fluorescent agent at a corresponding temperature; *R* is the gas constant, which is 8.314 J/mol K, and *T* is the reaction temperature.

By the slope and intercept of LogKa to 1/*T* linear graph, the values of $\Delta H^{○}$ and $\Delta S^{○}$ were determined; hence, the value of $\Delta G^{○}$ at the corresponding temperature was calculated.

### 3.10. Molecular Docking

The program AutoDock Vina was used to predict the type of interaction between synthetic peptides and pancreatic lipase. From the protein database (http://www.rcsb.org/pdb) (accessed on 20 May 2022), the crystal structure of pancreatic lipase (PDB ID:1ETH) was downloaded. Chem 3D was used to draw the 3D structure of the synthetic peptide; the charge by hydrogenation of the 3D structure of protein and synthetic peptide was calculated through AutoDock tools, and then the AutoDock Vina docking program was run. The docking parameters of the synthetic peptide and the whole pancreatic lipase we set as bellows: the central coordinates were x = 57.8, y = 44.8, z = 125.2, and the docking box size was x = 44, y = 30.0, z = 32.9, energy_ Range = 4, exhaustion = 10, Number modes = 10. The docking parameters of synthetic peptide and the active site of pancreatic lipase were set as the central coordinates: x = 72.1, y = 31.1, z = 144.7, the size of docking box: x = 92.6, y = 72.3, z = 145.7, energy_ range = 4, Exhaustiveness = 10, Number modes = 10. PyMOL was used to draw the three-dimensional interaction structure diagram of synthetic peptide and enzyme, and Discovery Studio 2016 was used to draw the two-dimensional diagram and the interaction type after docking was analyzed.

### 3.11. Lipid-Lowing Activity Evaluation

The HepG2 cells (Guangzhou Health Institute, Guangzhou, China) in logarithmic growth period were starved with serum-free medium for 12 h. Then, we replaced the medium with a complete medium containing 0.5 mM oleic acid and 0.4% FBS, and cultured this for 2–4 days (replacing the new oleic acid containing medium every day). The cells were divided into groups, and samples were added to each group as follows: Normal group: 1 mL of complete medium containing 0.4% serum; Model group: 1 mL of medium containing 0.5 mM oleic acid and 0.4% FBS; Positive control group: 1 mL of medium containing 2 mM orlistat, 0.5 mM oleic acid and 0.4% FBS; Sample treatment group: adding 1 mL of samples with different concentrations (high, medium, low) and medium containing 0.5 mM oleic acid and 0.4% FBS. After 2–4 days of culture, the cells in the Model group had a large amount of fat accumulation; 1 mL of PBS was washed 3 times and 4% paraformaldehyde solution was added and fixed at 37 °C for 1 h. Then, the paraformaldehyde solution was discarded, PBS was washed 3 times, and 1 mL of oil red O staining solution was added to each well. After dyeing at 37 °C for 1 h, the dye solution was discarded, washed with PBS 3 times, and 1 mL of PBS was added to prevent cell drying. The fat accumulation was observed under the inverted microscope and the photos were taken. According to the instructions of the triglyceride (TG) test box, the TG content of each well was measured using the GPO-PAP method, and the protein content was measured using the BCA method. The results were expressed as the triglyceride content per gram of protein (mmol/g protein).

### 3.12. Determination of Active Oxygen Content

Cells were cultured according to the above induction method, sucking the culture medium, washing twice with PBS, and adding 2 mL (10 μM) of DCFH-DA to each group. Then, they were incubated in a CO_2_ incubator (Froma series II, Thermo Scientific, Guangzhou, China) at 37 °C for 30 min, the DCFH-DA was sucked, and washed with PBS three times. Then, 0.5 mL of trypsin was added to digest for 1 min, followed by 1.5 mL of serum-free medium, and inhaled into a 2 mL centrifuge tube, before being centrifuged at 78 g for 10 min. Finally, the culture medium was poured, resuspended with PBS, and detected by flow cytometer.

### 3.13. Statistical Analysis

Each experiment was repeated at least 3 times. The data were given in the form of mean ± standard error, and the graph was drawn by Origin 2018. SPSS 20.0 was used to process the data, and the Ducan method was used to compare the significant difference, *p* < 0.05.

## Data Availability

Available upon request.

## Supplementary Materials

The following supporting information can be downloaded at: https://www.mdpi.com/article/10.3390/ijms24021529/s1.
