# Supplementary material for: Isolation and Identification of Lipid-Lowering Peptides from Sacha Inchi Meal
Source: Int J Mol Sci. 2023 Jan 12;24(2):1529. doi: 10.3390/ijms24021529 (PMC9863159; doi:10.3390/ijms24021529)
Supplement: Supplementary file 1 [file ijms-24-01529-s001.zip › ijms-2115480-supplementary.pdf]

Supplemental materials

Table S1. Kinetic parameters of pancreatic lipase at different concentrations of synthetic peptides.

Peptides	V_{max} (FLU/ms)	K_m (mM)	K_i ($K_{i'}$) (μM·ms^2·FLU^{-2})	Type
NV-7	4.38±1.54	1.65±0.07	77.33±7.65 (7.80±0.77)	Mixed
WK-5	1.32±0.01	0.67±0.01	577.60±25.69 (1225.28±55.20)	Mixed
WK-8	1.12±0.04	0.46±0.02	191.91±25.33	Non-competitive
EY-8	0.47±0.06	0.15±2.71×10^{-3}	578.30±58.12 (161.47±16.22)	Mixed
FK-9	0.48	0.21±0.01	80.94±2.85	Competitive

Table S2. Quenching constants K_{sv}, binding constants K_a and relative thermodynamic parameters of the interaction between interaction synthetic peptides and pancreatic lipase (PL) at different temperatures.

Components	Temperature (K)	K_{sv} (×10^3 L·mol^{-1})	R_a^2	K_a (×10^3 L·mol^{-1})	n	R_b^2	ΔH° (KJ·mol^{-1})	ΔS° (J·mol^{-1})	ΔG° (KJ·mol^{-1})
NV-7-PL	298	4.62±0.25	0.9999	10.66	1.43±0.15	0.9469	-49.25	-87.43	-23.20
	304	3.92±0.23	0.9999	9.41	1.40±0.17	0.9332			-22.68
	310	3.27±0.22	0.9999	4.92	1.07±0.13	0.9290			-22.15
FK-9-PL	298	6.95±0.42	0.9993	3.56	0.67±0.04	0.9817	20.31	135.97	-20. 21
	304	7.79±0.41	0.9993	3.92	0.66±0.04	0.9798			-21.02
	310	11.09±0.67	0.9985	4.89	0.56±0.04	0.9716			-21.84

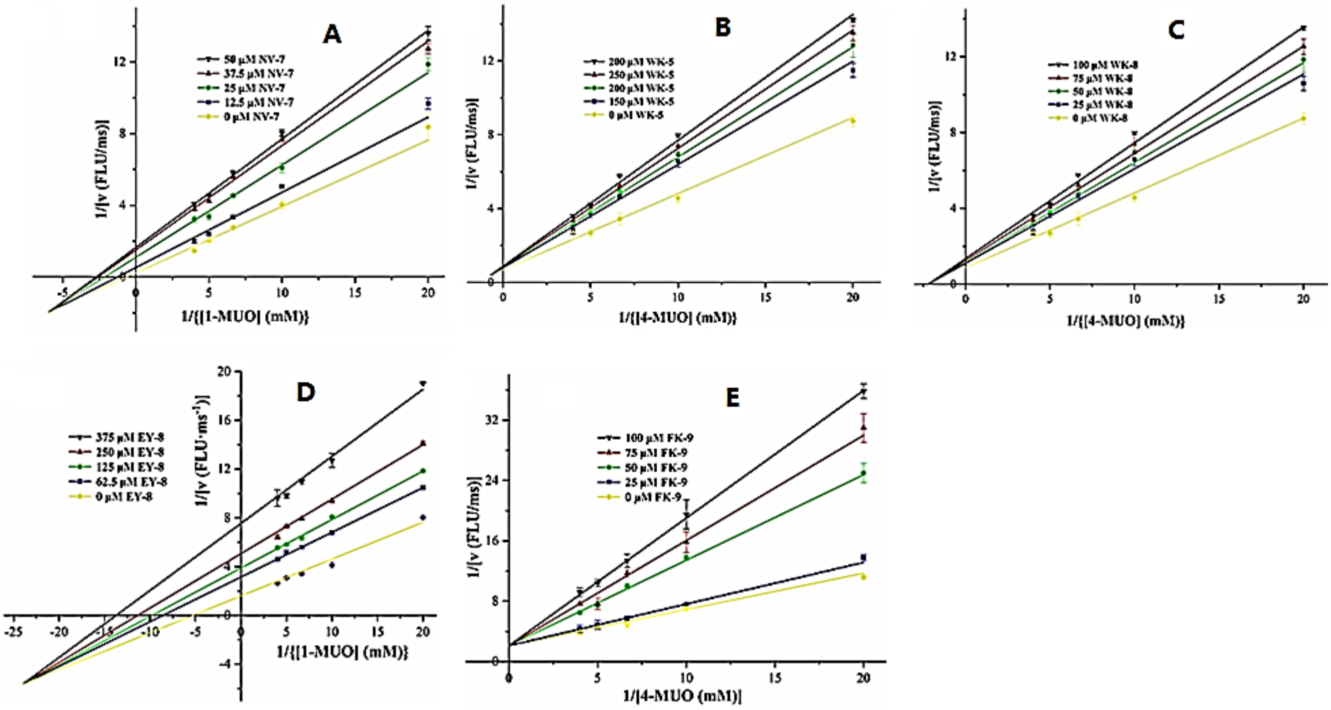

Figure S1. Lineweaver-Burk plots of pancreatic lipase substrate concentration and reaction rate at different concentrations of synthetic peptides (A) NV-7, (B) WK-5, (C) WK-8, (D) EY-8, (E) FK-9.

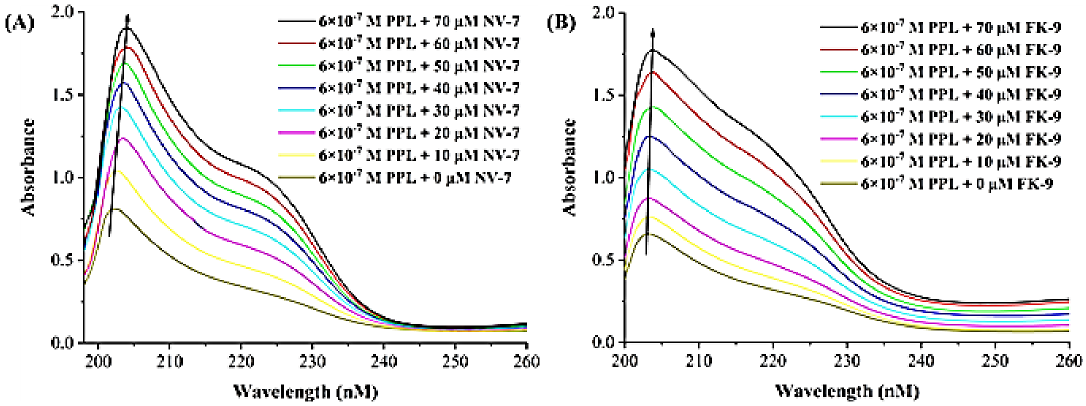

Figure S2. Analysis of the interaction between synthetic peptides and pancreatic lipase by UV spectra.

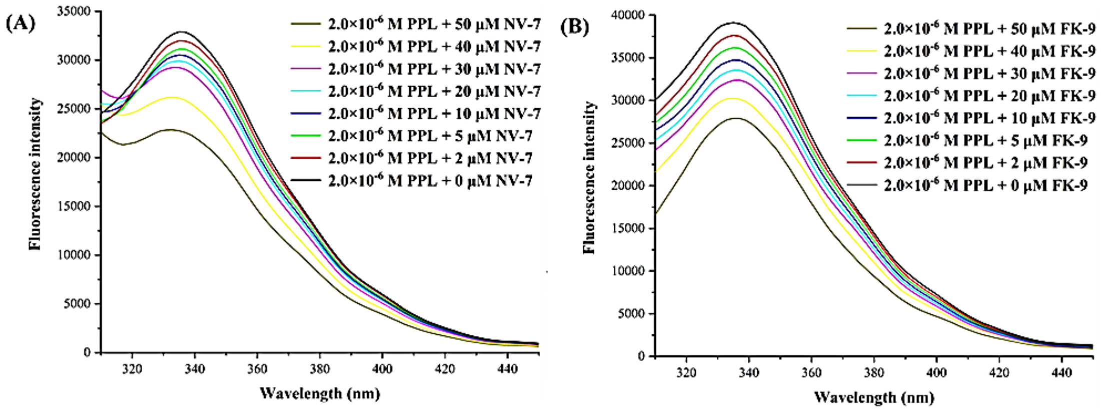

Figure S3. Quenching effects of synthetic peptides on the fluorescence spectra of pancreatic lipase.
